# Suitability for Transcarotid Transcatheter Aortic Valve Replacement in the Japanese Population

**DOI:** 10.1016/j.jacasi.2025.10.018

**Published:** 2026-03-03

**Authors:** Masaki Nakashima, Natsuko Satomi, Daishi Tazawa, Momo Kosuga, Manabu Maeda, Yun Teng, Yuta Kobayashi, Makoto Saigan, Yusuke Toki, Yusuke Enta, Masaki Miyasaka, Yoshiko Munehisa, Akihiro Yamamoto, Norio Tada

**Affiliations:** a Department of Cardiology, Sendai Kousei Hospital, Miyagi, Japan; b Department of Cardiovascular Medicine, Faculty of Medicine and Graduate School of Medicine, Hokkaido University, Sapporo, Japan; c Department of Laboratory Medicine, The Jikei University School of Medicine, Tokyo, Japan; d Department of Cardiovascular Surgery, Sendai Kousei Hospital, Miyagi, Japan

**Keywords:** aortic valve stenosis, atherosclerosis, common carotid artery, heart valve prosthesis implantation, ischemic heart disease, vascular complications

## Abstract

**Background:**

Transcarotid (TC) transcatheter aortic valve replacement (TAVR) has shown favorable outcomes in selected Western populations; however, data on its suitability in patients undergoing TAVR remain scarce.

**Objectives:**

The purpose of this study was to investigate the anatomical characteristics of the common carotid artery (CCA) and the suitability of Japanese patients for TC-TAVR.

**Methods:**

This single-center retrospective study included consecutive patients who underwent TAVR between April 2023 and March 2024. Patients were categorized into a tough-transfemoral (TF) group, who may require an alternative approach, and a viable-TF group. TC-TAVR suitability was determined based on the CCA diameter, exposure site calcification, atherosclerotic plaque, and contralateral carotid artery stenosis.

**Results:**

Among 336 patients, the right CCA was larger than the left CCA (6.6 ± 1.0 mm vs 6.4 ± 0.9 mm; *P* < 0.001) but exhibited greater tortuosity (44 of 336 [13.1%; 95% CI: 9.9%-17.1%] vs 9 of 336 [2.7%; 95% CI: 1.4%-5.0%]; *P* < 0.001). The tough-TF group had a higher incidence of major vascular complications (12 of 117 [10.3%; 95% CI: 6.0%-17.1%]) compared with the viable-TF group (3 of 219 [1.4%; 95% CI: 0.5%-3.9%]; *P* < 0.001). Overall suitability for TC-TAVR was high (306 of 336 [91.1%; 95% CI: 87.5%-93.7%]); however, it was significantly lower in the tough-TF group than in the viable-TF group (101 of 117 [86.3%; 95% CI: 78.9%-91.4%] vs 205 of 219 [93.6%; 95% CI: 89.6%-96.2%]; *P =* 0.042). Multivariate regression analysis identified peripheral artery disease as a negative predictor of suitability (OR: 0.42; 95% CI: 0.19-0.99; *P =* 0.039).

**Conclusions:**

Although TC-TAVR suitability was compromised in the tough-TF group, 86% remained suitable, suggesting that TC-TAVR may serve as a promising approach in the Japanese population.

The gold standard for transcatheter aortic valve replacement (TAVR) is the transfemoral (TF) approach, whereas 5% to 10% of patients require an alternative route because of anatomical limitations.^1^^,^^2^ Among the alternative access methods, reports from the TVT registry have shown a shift away from central forms (eg, transapical and direct-aortic) toward more peripheral alternatives (eg, trans-subclavian).^3^

Transcarotid (TC) TAVR was first reported in 2016 as a novel alternative for patients with contraindications to other approaches.^4^ It has alleviated the disadvantages of alternative access by offering reduced invasiveness and improved clinical outcomes.^5^^,^^6^ Furthermore, TC-TAVR has demonstrated 30-day efficacy and safety comparable to TF-TAVR, leading to its recognition as a promising alternative.^7^ Reflecting these findings, TC-TAVR was reimbursed in Japan in May 2024. Additionally, a consensus statement on alternative access for TAVR from the Society for Cardiovascular Angiography and Interventions recommended the TC or transcaval approach over other alternative routes when anatomically feasible.^8^

Feasibility for TC-TAVR is mainly affected by the anatomy of the common carotid artery (CCA). Although the TC-TAVR has been performed in selected Western populations, there are no previous reports investigating TC-TAVR suitability in the broader TAVR population. Particularly in Japanese patients, who generally have a smaller body size, suitability remains unclear. This study aimed to examine the anatomical features of the CCA, such as diameter, tortuosity, calcification, and atherosclerotic plaque severity. Right-to-left comparisons were performed to identify potential differences. TC-TAVR suitability was also compared between 2 groups: one with complex anatomical or procedural features for TF-TAVR and another with viable indications for TF-TAVR.

## Methods

### Study design and population

This retrospective, observational, single-center study enrolled consecutive patients with severe aortic stenosis who underwent TAVR at Sendai Kosei Hospital (Miyagi, Japan) between April 2023 and March 2024. Indications for TAVR were determined based on a comprehensive evaluation of clinical, anatomical, and patient-related factors.^9^ To evaluate a broad spectrum of patient backgrounds, we included not only first-time TAVR cases with native tricuspid aortic valves, but also patients with congenital bicuspid valves, TAVR-in-surgical aortic valve replacement, and TAVR-in-TAVR procedures. Patients without preprocedural contrast-enhanced computed tomography (ceCT) were excluded from the analysis. The study conformed to the principles outlined in the Declaration of Helsinki and was approved by the Institutional Review Board of the Sendai Kosei Hospital Clinical Research Ethics Committee (approval number: 6-48).

### TAVR strategy and procedural endpoint

The TAVR approach, along with the type and size of the transcatheter heart valve and type of anesthesia, was assessed by a local heart team. Self-expandable valves (SEVs), such as the Evolut FX (Medtronic) and NAVITOR (Abbott), were approved for TF, subclavian, and direct-aortic access. The balloon-expandable valve (BEV), SAPIEN 3 Ultra Resilia (Edwards Lifesciences), was additionally approved for transapical access. However, TC-TAVR was not approved for use in Japan during the study period.

TF-TAVR was performed as the first-line approach, using ultrasound-guided common femoral artery puncture and preclosure using the two-Perclose (Abbott) technique. Other TAVR procedures employed surgical cut-downs. The procedural endpoint was based on vascular complication (VC), evaluated according to the criteria established by the Valve Academic Research Consortium-3 (VARC-3).^10^

### CT acquisition and assessment

Vascular assessment was performed using non-electrocardiogram-gated ceCT with a 320-detector row computed tomography (CT) scanner (Aquilion ONE, INSIGHT edition; Toshiba Medical Systems). Image reconstruction and measurement were conducted with a slice thickness of 0.5 mm using a Revoras workstation (version 5.2.1.1, Ziosoft) or Synapse viewer (version 5.5.000V5.1, Fujifilm Corporation). The iliofemoral artery lumen diameter was measured using curved multiplanar reconstruction images, whereas the carotid artery diameters were measured using axial images. For qualitative assessments of vascular calcification, tortuosity, and morphological characteristics, a combination of 3-dimensional reconstruction and axial imaging was utilized.

### TF access assessment

To assess TF access complexity, we established criteria combining known risk factors with potential procedural complexity modifiers. The tough-TF group was defined by the presence of any of the following criteria: 1) bilateral iliofemoral artery minimum vessel diameter ≤5 mm; 2) heavily, defined as grade 2 or 3, calcified iliac artery (0 = none, no calcification; 1 = mild, <30% of the vessel was covered by calcium; 2 = moderate, 30% to 60%; and 3 = severe, 60% to 100%)^11^; 3) tortuous aorta^12^; 4) shaggy aorta (defined as plaque thickness >3 mm)^13^^,^^14^; 5) prior aortic intervention or presence of aortic aneurysm; 6) prior iliac stenting; 7) use of a 65-cm DrySeal sheath (W.L. Gore and Associates); or 8) iliac artery balloon dilatation for device passage. Patients with unilateral lesions and a viable contralateral side were not classified into the tough-TF group. Patients who did not meet any of the criteria were classified into the viable-TF group.

### Evaluation of the CCA

The diameter and calcification of the CCA were measured at the surgical exposure site, located low in the neck at the level of the omohyoid muscle, and at the proximal minimal lumen diameter, respectively. There is currently no established classification system for the quantitative evaluation of calcification or plaque in the CCA. Therefore, we adopted criteria originally developed for the iliofemoral arteries^12^ and the aorta, in which plaque was defined as a thickness >3 mm.^13^^,^^14^ Vascular tortuosity was categorized as straight, tortuous, kinking, or coiling, based on a previous study.^15^ We also classified aortic arch morphology.

### Suitability for TC-TAVR

The right and left CCAs were evaluated separately for TC-TAVR suitability. TC-TAVR via the ipsilateral CCA was considered suitable based on the following criteria: 1) minimum required CCA diameter; 2) calcification of grade 0 or 1 at the surgical exposure site; 3) absence of atherosclerotic plaque; and 4) no contralateral carotid artery stenosis >50% on echography. The minimum required CCA diameter was determined as follows: in cases where BEVs were used, a threshold ≥5.5 mm was applied for 20-, 23-, and 26-mm valves, while a threshold ≥6.0 mm was applied for 29-mm valves. In cases using SEVs, a threshold ≥5.5 mm was applied for annulus area ≤540 mm^2^, and ≥6.0 mm when it exceeded 540 mm^2^, assuming BEV size was selected based on annulus area.^8^ CCA tortuosity and aortic arch variants were not taken into consideration. Cerebral magnetic resonance imaging was excluded from the analysis caused by limited availability (81 of 366 [22%]).

### Statistical analyses

Categorical variables were reported as absolute values and percentages. Continuous variables are expressed as mean ± SD when normally distributed and as median (IQR) when not normally distributed. Categorical variables were compared using the chi-square test or Fisher exact test, as appropriate. The Student’s *t-*test was used to compare normally distributed continuous variables, and the Mann-Whitney *U* test was used for non-normally distributed continuous variables. For right–left carotid artery comparisons, Wilcoxon signed-rank tests were used for continuous variables and McNemar's tests or McNemar-Bowker tests for categorical variables according to the table size. Incidence outcome rates are presented with 95% CIs, which were estimated using Wilson’s method for binomial proportions.

Logistic regression analysis was performed to identify clinical predictors of TC-TAVR suitability. For the multivariate analysis, covariates were prespecified a priori based on clinical relevance to carotid anatomy and TC-TAVR suitability according to prior literature. Given the limited number of events, we specified a parsimonious primary multivariable logistic regression model including age, sex, body surface area, hypertension, and peripheral artery disease (PAD). Continuous variables were modeled linearly (age per 1 year; body surface area per 0.1 m^2^). Sensitivity analyses assessed robustness by additionally adjusting for ischemic heart disease (IHD) or prior cerebrovascular accident (one at a time), as well as by including chronic kidney disease (CKD) or by replacing CKD with hemodialysis. As there were no missing values, all models included 336 patients. To address small-sample bias and potential separation, we also performed Firth’s penalized logistic regression with the same covariates as the primary model. Collinearity was assessed using variance inflation factors. Two-sided *P <* 0.05 was considered statistically significant. All statistical analyses were performed using R version 4.4.2 (The R Foundation for Statistical Computing).

## Results

### Study population, TF assessment, and procedural outcome

**Figure 1:**
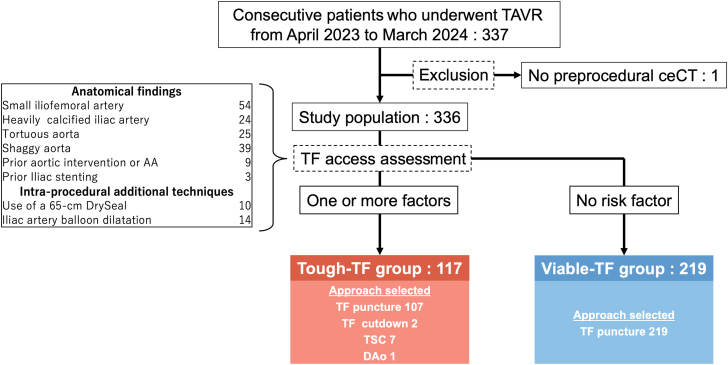
Patient Flowchart Patients were categorized into the tough- or viable-transfemoral (TF) groups based on preprocedural and intraprocedural factors. AA = aortic aneurysm; ceCT = contrast-enhanced computed tomography; DAo = direct-aortic; TAVR = transcatheter aortic valve replacement; TSC = trans-subclavian.

A total of 337 patients underwent TAVR during the study period. One patient was excluded because of the absence of preprocedural ceCT. Of the remaining 336 patients, 117 of 336 (35%) were categorized into the tough-TF group based on the assessment of TF access. In this group, 107 of 117 (91%) patients underwent TAVR via the TF puncture method. The combinations of anatomical findings, intraprocedural additional techniques, and access strategies are presented in Supplemental Table 1. Each anatomical factor was associated with the need for a DrySeal sheath, balloon dilatation, or alternative access (Supplemental Figure 1). In the viable-TF group, all patients underwent TF-TAVR via the TF puncture method (Figure 1).

Table 1 presents the characteristics of the patients in the tough- and viable-TF groups. The average age was 82.6 ± 6.5 years, and 164 of 336 (49%) patients were men. Patients had a median Society of Thoracic Surgeons Predicted Risk of Mortality (STS-PROM) score of 7.4 (Q1-Q3: 4.7-13.2), reflecting an elevated operative risk in this cohort. Notably, 61 of 336 (18%) patients underwent hemodialysis. The tough-TF group was associated with small body size, higher STS-PROM score, higher NYHA functional class, PAD, IHD, and reduced left ventricular ejection fraction (all *P <* 0.050). The size of the aortic annulus was similar between the groups.

**Table 1:** Patient Characteristics

	Overall (N = 336)	Tough TF (n = 117)	Viable TF (n = 219)	*P* Value
Clinical findings				
Age, y	82.6 ± 6.5	83.5 ± 5.9	82.1 ± 6.8	0.052
Male	164 (48.8)	58 (49.6)	106 (48.4)	0.928
Height, m	154 ± 10	153 ± 11	155 ± 10	0.031
Body weight, kg	54.1 ± 11.4	50.8 ± 10.1	55.8 ± 11.7	<0.001
BMI, kg/m^2^	22.6 ± 3.8	21.7 ± 3.3	23.1 ± 3.9	<0.001
BSA, m^2^	1.5 (1.4-1.6)	1.5 (1.3-1.6)	1.5 (1.4-1.7)	0.002
CFS ≧6	40 (11.9)	18 (15.4)	22 (10.0)	0.207
NYHA functional class III or IV	97 (28.9)	43 (36.8)	54 (24.7)	0.028
STS-PROM	7.4 (4.7-13.2)	10.3 (5.9-16.6)	6.1 (4.0-10.4)	<0.001
Hypertension	271 (80.7)	94 (80.3)	177 (80.8)	1.000
Dyslipidemia	179 (53.3)	61 (52.1)	118 (53.9)	0.849
Diabetes mellitus	97 (28.9)	37 (31.6)	60 (27.4)	0.491
Atrial fibrillation	93 (27.7)	39 (33.3)	54 (24.7)	0.118
PAD	63 (18.8)	33 (28.2)	30 (13.7)	0.002
CKD	264 (78.6)	97 (82.9)	167 (76.3)	0.157
On hemodialysis	61 (18.2)	24 (20.5)	37 (16.9)	0.502
Smoke	19 (5.7)	10 (8.5)	9 (4.1)	0.153
Prior ischemic heart disease	109 (32.4)	49 (41.9)	60 (27.4)	0.010
Prior cerebral vascular accident	31 (9.2)	14 (12.0)	17 (7.8)	0.284
Chronic respiratory failure	83 (24.7)	30 (25.6)	53 (24.2)	0.874
Bicuspid aortic valve	23 (6.8)	4 (3.4)	19 (8.7)	0.112
TAVR in SAVR	14 (4.2)	6 (5.1)	8 (3.7)	0.571
TAVR in TAVR	3 (0.9)	1 (0.9)	2 (0.9)	1.000
Echocardiography data				
LVEF ,%	54.9 ± 11.4	52.8 ± 13.1	55.9 ± 10.3	0.044
LVDd, mm	45.0 ± 6.4	44.1 ± 5.5	45.4 ± 6.7	0.128
Aortic valve mean gradient, mm Hg	48.5 ± 18.9	48.0 ± 20.0	48.8 ± 18.4	0.756
Aortic valve area, cm^2^	0.76 (0.62-0.87)	0.72 (0.60-0.82)	0.78 (0.63-0.87)	0.123
CT measurement				
Annulus area, mm	435.6 ± 88.7	426.1 ± 82.8	440.7 ± 91.5	0.149
Annulus area >540 mm^2^	41 (12.2)	31 (14.2)	10 (8.5)	0.186
Annulus perimeter, mm	74.8 ± 7.8	74.0 ± 7.5	75.3 ± 7.9	0.142
Ascending aorta, mm	33.4 ± 3.8	33.6 ± 3.4	33.4 ± 4.1	0.555
Aortic root angle	48.7 ± 9.4	50.0 ± 9.4	48.0 ± 9.4	0.069

Values are mean ± SD, median (Q1-Q3), or n (%).

BMI = body mass index; BSA = body surface area; CFS = clinical frailty scale; CKD = chronic kidney disease; CT = computed tomography; LVDd = left ventricular end-diastolic diameter; LVEF = left ventricular ejection fraction; PAD = peripheral artery disease; SAVR = surgical aortic valve replacement; STS-PROM = Society of Thoracic Surgeons predicted risk of mortality; TAVR = transcatheter aortic valve replacement; TF = transfemoral.

Approximately 70% of patients underwent general anesthesia. Procedural time, fluoroscopy time, and contrast volume were higher in the tough-TF group. VCs were more common in this group. Notably, major VCs occurred in 12 of 117 (10.3%; 95% CI: 6.0%-17.1%) patients in the tough-TF group compared with 3 of 219 (1.4%; 95% CI: 0.5%-3.9%) in the viable-TF group (*P <* 0.001). Although VCs in the viable-TF group were primarily limited to device-closure failure, the tough-TF group exhibited a broader spectrum of complications, including aortic rupture/dissection, vascular injury, unplanned interventions, and distal embolization (Table 2). Both procedural and fluoroscopy times were significantly long in the tough-TF group regardless of VCs. In cases with VCs, all procedural indicators increased further in the tough-TF group (all *P <* 0.05) (Supplemental Figures 2 and 3).

**Table 2:** Procedure Indicators and Vascular Complications

	Overall (N = 336)	Tough TF (n = 117)	Viable TF (n = 219)	*P* Value
Type of anesthesia				
General anesthesia	238 (70.8)	157 (71.7)	81 (69.2)	0.7290
Local sedation	98 (29.2)	62 (28.3)	36 (30.8)	
Access site conversion	0	0	0	
Periprocedural variables				
Anesthesia time, min	111 ± 38	127 ± 50	103 ± 26	<0.001
Procedure time, min	66 ± 35	78 ± 48	59 ± 23	<0.001
Fluoroscopy time, min	26 ± 15	31 ± 20	23 ± 11	<0.001
Contrast volume, mL	111 ± 50	123 ± 55	105 ± 47	<0.001
Any vascular complications	31 (9.2)	19 (16.2)	12 (5.5)	0.002
Major vascular complications	15 (4.5)	12 (10.3)	3 (1.4)	<0.001
Aortic rupture/dissection	5 (1.5)	4 (3.4)	1 (0.5)	
Vascular injury	3 (0.9)	3 (2.6)	0 (0)	
Unplanned intervention	4 (1.2)	4 (3.4)	0 (0)	
Distal embolization	2 (0.6)	2 (1.7)	0 (0)	
Device closure failure	4 (1.2)	2 (1.7)	2 (0.9)	
Minor vascular complications	19 (5.7)	9 (7.7)	9 (4.1)	0.256
Vascular injury	9 (2.7)	5 (4.3)	4 (1.8)	
Unplanned intervention	4 (1.2)	4 (3.4)	0 (0)	
Distal embolization	0 (0)	0 (0)	0 (0)	
Device closure failure	6 (1.8)	1 (0.9)	5 (2.3)	

Values are mean ± SD or n (%). The table includes duplicate items.

TF = transfemoral.

### Anatomical characteristics of the CCA and aortic arch

**Figure 2:**
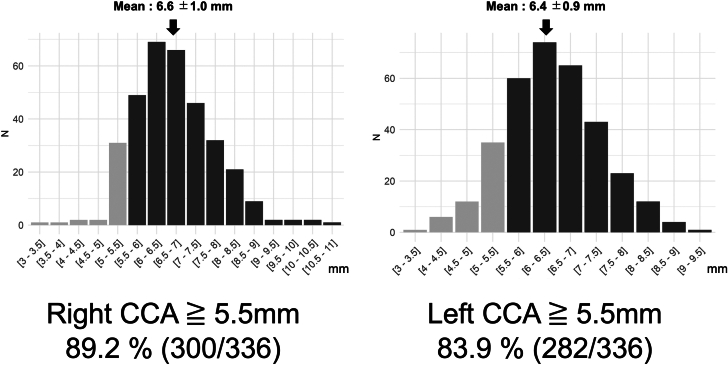
Distribution of Minimal CCA Diameter Histograms illustrate the minimal diameters of the right and left common carotid arteries (CCAs). Light gray bars indicate diameters <5.5 mm, and dark bars indicate diameters ≥5.5 mm. The mean minimal diameter was 6.6 ± 1.0 mm on the right and 6.4 ± 0.9 mm on the left; the right side was significantly larger (*P <* 0.001). CCA = common carotid artery.

The distributions of the right and left minimal CCA diameters are shown in Figure 2. The right CCA diameter was significantly larger than the left (6.6 ± 1.0 mm vs 6.4 ± 0.9 mm; *P* < 0.001). A minimum CCA diameter ≥5.5 mm was observed in 300 of 336 (89%; 95% CI: 85.5%-92.2%) and 282 of 336 (84%; 95% CI: 79.6%-87.5%) of patients on the right and left sides, respectively. The 5.5-mm threshold was adopted as only 22 of 336 (6.5%; 95% CI: 4.4%-9.7%) of all cases (20 for BEV and 2 for SEV) required a minimum vessel diameter of 6.0 mm (Supplemental Table 2).

The characteristics of the carotid arteries and aortic arch are summarized in Table 3. The right CCA was approximately 0.3 mm larger than the left across all measured sites (*P <* 0.001). Calcification at the narrowest segment tended to be more severe on the right side *(P =* 0.003). Severe tortuosity of the CCA was significantly more pronounced on the right side, with 30 of 336 (8.9%; 95% CI: 6.3%-12.5%) exhibiting kinking and 14 of 336 (4.2%; 95% CI: 2.5%-6.9%) coiling (*P <* 0.001). Among aortic arch anomalies, a common origin of the brachiocephalic artery and left CCA was observed in 25 of 336 (7.4%; 95% CI: 5.1%-10.8%) of cases, a bovine aortic arch in 16 of 336 (4.8%; 95% CI: 3.0%-7.6%), and 2 other cases, namely 1 postoperative state of the aortic arch and 1 arteria lusoria, were observed.

**Table 3:** Characteristics of Carotid Arteries and Aortic Arch

	Right CCA	Left CCA	Difference^a^ (95% CI)	*P* Value
At surgical exposure site				
Maximum diameter	7.6 ± 1.1	7.3 ± 1.1	0.35 (0.25-0.45)	<0.001
Minimum diameter	7.0 ± 1.1	6.7 ± 1.0	0.30 (0.25-0.40)	<0.001
Degree of calcification				
None	284 (85)	279 (83)		0.217
Mild	44 (13)	44 (13)		
Moderate	6 (1.8)	13 (3.9)		
Severe	2 (0.6)	0		
At minimal lumen site				
Maximum diameter	7.5 ± 1.2	7.2 ± 1.1	0.30 (0.20-0.40)	<0.001
Minimum diameter	6.6 ± 1.0	6.4 ± 0.9	0.25 (0.20-0.35)	<0.001
Degree of calcification				
None	227 (68)	255 (76)		0.003
Mild	99 (29)	72 (21)		
Moderate	10 (3.0)	9 (2.7)		
Severe	0	0		
Atherosclerotic plaque	20 (6.0)	12 (3.6)		0.170
Tortuosity				
Straight	144 (43)	244 (73)		<0.001
Tortuosity	148 (44)	83 (9.1)		
Kinking	30 (8.9)	4 (1.2)		
Coiling	14 (4.2)	5 (1.5)		
Aortic arch branching				
Normal	293 (87)			
Common origin of BCA/LCCA	25 (7.4)			
Bovine	16 (4.8)			
Others	2 (0.6)			

Values are mean ± SD or n (%).

BCA = brachiocephalic artery; CCA = common carotid artery; LCCA = left common carotid artery.

a The difference represents right minus left.

### Suitability for TC-TAVR

Of the 336 patients, 306 (91%; 95% CI: 87.5%-93.7%) were suitable for TC-TAVR on at least 1 side, and 221 (66%; 95% CI: 60.5%-70.6%) were suitable on both sides. TC-TAVR suitability was evenly distributed between the right and left sides (Supplemental Figure 4). The primary reason for unsuitability was a small CCA diameter, occurring in 39 of 336 (12%; 95% CI: 8.6%-15.5%) on the right and 56 of 336 (17%; 95% CI: 13.1%-21.0%) on the left *(P =* 0.076) (Supplemental Figure 5).

In univariate analyses, PAD was the only negative predictor of TC-TAVR suitability (OR: 0.42; 95% CI: 0.19-0.98; *P =* 0.036). In the primary multivariable logistic regression model adjusting for age, sex, body surface area, and hypertension, PAD remained an independent negative predictor of TC-TAVR suitability (OR: 0.42; 95% CI: 0.19-0.99; *P =* 0.039) (Table 4). Results were materially unchanged when using Firth’s penalized logistic regression with the same covariates (OR: 0.41; 95% CI: 0.19-0.96; *P =* 0.040). No concerning multicollinearity was observed; all variance inflation factor values were <2. Sensitivity analyses yielded consistent findings. The association of PAD with lower TC-TAVR suitability was robust to additional adjustment for CKD or hemodialysis. Adjustment for IHD or prior cerebrovascular accident attenuated the statistical significance, but the effect direction was unchanged (Supplemental Table 3).

**Table 4:** Predictors for Transcarotid Transcatheter Aortic Valve Replacement Eligibility by Univariate and Multivariate Regression Analysis

	Univariate Analysis			Multivariate Analysis		
	OR	95% CI	*P* Value	OR	95% CI	*P* Value
Age (per 1 y)	0.99	0.93-1.05	0.737	1.00	0.93–1.06	0.931
Male	1.10	0.52-2.36	0.806	0.87	0.33–2.34	0.786
BSA (per 0.1 m^2^)	1.11	0.91-1.36	0.330	1.12	0.84–1.50	0.459
CFS ≧6	0.87	0.31-3.06	0.800			
NYHA functional class III or IV	0.79	0.36-1.83	0.572			
STS-PROM	0.98	0.94-1.02	0.204			
Hypertension	1.91	0.80-4.29	0.127	1.86	0.76–4.24	0.150
Dyslipidemia	0.74	0.34-1.57	0.440			
Diabetes mellitus	0.79	0.36-1.83	0.572			
Atrial fibrillation	1.06	0.47-2.61	0.897			
PAD	0.42	0.19-0.98	0.036	0.42	0.19–0.99	0.039
CKD	1.37	0.55-3.12	0.465			
Hemodialysis	1.49	0.55-5.19	0.475			
Smoke	0.82	0.22-5.36	0.802			
Prior ischemic heart disease	0.70	0.33-1.54	0.356			
Prior cerebrovascular accident	0.63	0.22-2.25	0.419			
Chronic respiratory failure	1.09	0.47-2.83	0.855			

Abbreviations as in Table 1.

### Suitability for TC-TAVR between the tough- and viable-TF groups

**Figure 3:**
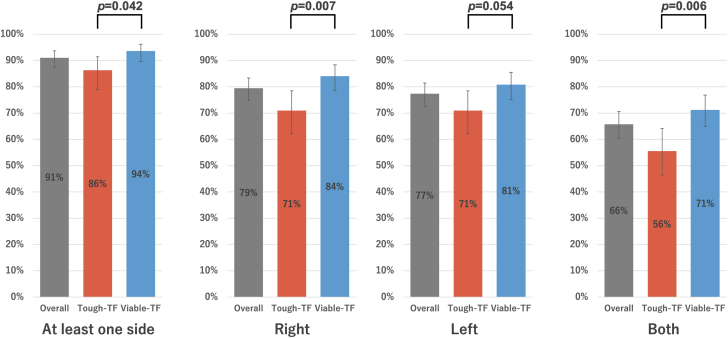
TC-TAVR Suitability by Side and Group Of the 336 patients, 306 (91%) were suitable for transcarotid transcatheter aortic valve replacement on at least 1 side. Suitability was lower in the tough-transfemoral (TF) group than in the viable-TF groups (86% vs 94%; *P =* 0.042), driven by a higher rate of right-side unsuitability in the tough-TF group (71% vs 84%; *P =* 0.007). TF = transfemoral.

**Figure 4:**
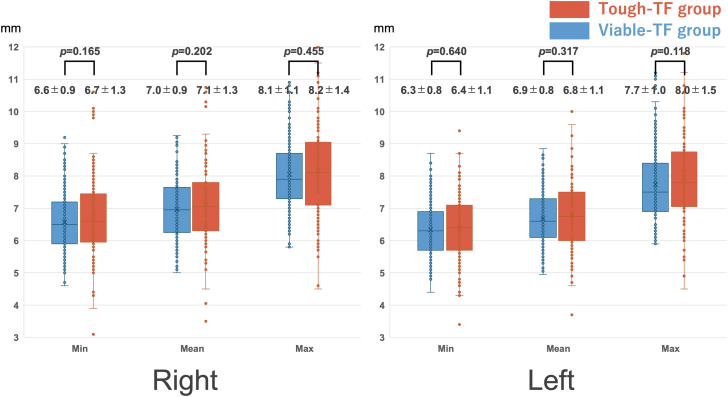
Common Carotid Artery Diameters by Side and Group Box plots compare the minimal, mean, and maximal common carotid artery diameters by side and access group. The right common carotid artery tended to be larger than the left in both groups, and no significant differences were observed between groups for any measures. TF = transfemoral.

**Figure 5:**
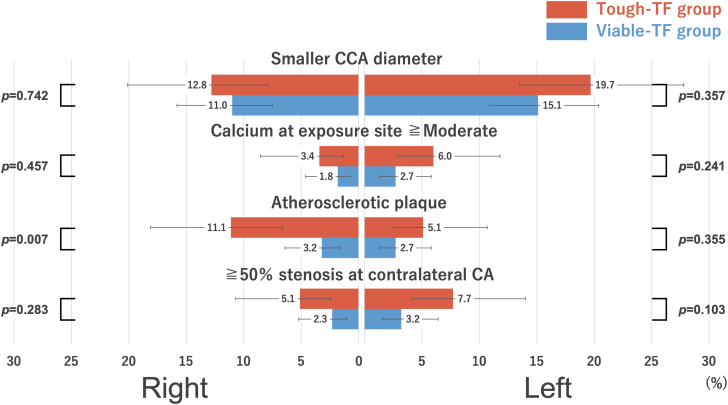
Reasons for Transcarotid Transcatheter Aortic Valve Replacement Unsuitability by Group A smaller vessel diameter was the primary cause of unsuitability in both groups. In the tough-transfemoral (TF) group, right-sided access was more often limited by atherosclerotic plaque (11.1% vs 3.2%; *P =* 0.007), whereas the left-sided access was more frequently affected by contralateral carotid artery disease (7.7% vs 3.2%; *P =* 0.103). CA = carotid artery; CCA = common carotid artery.

**Figure 6:**
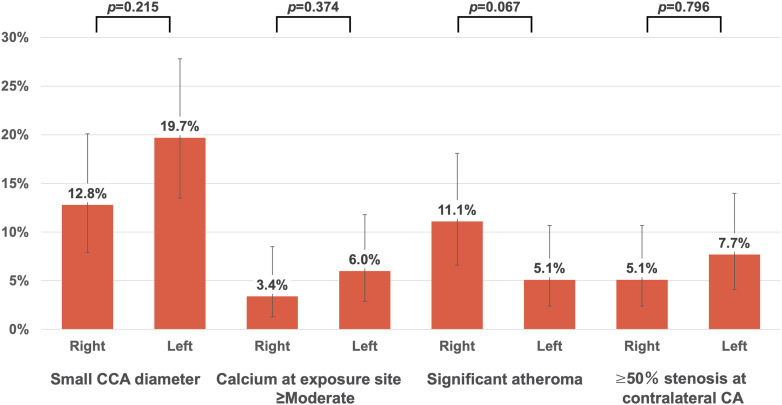
Reasons for Transcarotid Transcatheter Aortic Valve Replacement Unsuitability in the Tough-Transfemoral Group In the tough-transfemoral group, small vessel diameters were more common on the left side, and atherosclerotic plaque was more common on the right; however, neither difference reached statistical significance. Abbreviations as in Figure 5.

Overall suitability for TC-TAVR was lower in the tough-TF (101 of 117 [86%; 95% CI: 78.9%-91.4%]) than the viable-TF group (205 of 219 [94%; 95% CI: 89.6%-96.2%]; *P =* 0.042), attributed to the higher frequency of right-side unsuitability in the tough-TF group (83 of 117 [71%; 95% CI: 62.2%-78.4%] vs 184 of 219 [84%; 95% CI: 78.6%-88.3%]; *P =* 0.007) (Figure 3). Although not statistically significant, the CCA diameter in the tough-TF group was slightly larger than in the viable-TF group (Figure 4). When comparing the reasons for unsuitability between the groups, contributions of CCA diameter and calcification were similar. However, atherosclerotic plaques were more frequently observed on the right side (13 of 117 [11.%; 95% CI: 6.6%-18.1%] vs 7 of 219 [3.2%; 95% CI: 1.6%-6.4%]; *P =* 0.007), while contralateral carotid artery stenosis or occlusion was more common on the left side (9 of 117 [7.7%; 95% CI: 4.1%-14.0%] vs 7 of 219 [3.2%; 95% CI: 1.6%-6.4%]), although not statistically significant *(P =* 0.103) (Figure 5). In the tough-TF group, although not statistically significant, the right CCA tended to be larger and more frequently associated with atherosclerotic plaques than the left CCA (Figure 6).

## Discussion

### Principal findings

This study is the first to evaluate the anatomical characteristics of the CCA and TC-TAVR suitability in patients undergoing TAVR. Overall, 91% of patients and 86% of those in the tough-TF group were suitable for TC-TAVR. When comparing sides, right and left CCAs were equally suitable. Despite its larger diameter, right-sided TC-TAVR was more frequently deemed unsuitable in the tough-TF group because of atherosclerotic plaques. Among clinical predictors, PAD emerged as the only independent factor associated with TC-TAVR unsuitability. Given these findings, optimizing access selection in the tough-TF group has the potential to improve procedural outcomes, with TC-TAVR serving as a viable alternative approach not only in Western countries but also in Asian populations (Central Illustration).

**Central Illustration:**
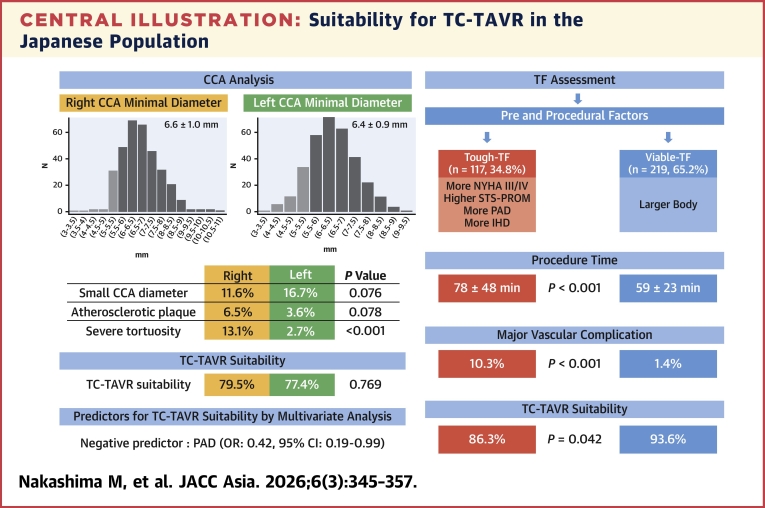
Suitability for TC-TAVR in the Japanese Population This cohort comprised 336 transcatheter aortic valve replacement (TAVR) patients. Although right common carotid artery (CCA) was larger; it exhibited greater tortuosity than the left. Tough-transfemoral (TF) access was identified in 117 patients (35%), and was associated with longer procedure times and a higher incidence of major vascular complications. Despite lower overall TC-TAVR suitability compared with the viable-TF group, 86% of patients in the tough-TF group remained suitable. IHD = ischemic heart disease; PAD = peripheral artery disease; STS-PROM, Society of Thoracic Surgeons-Predicted Risk of Mortality; TC = transcarotid.

### Assessment of TF and TC access in the study population

The TF puncture technique has been widely applied because of its minimally invasive nature; however, VCs remain a major concern, even in modern low-profile TAVR systems. Recent randomized controlled trials have reported a 2.0% to 3.8% incidence of major VC.^16^^,^^17^ In the Asian populations, the incidence was 2.48% ± 2.54%.^18^ Known risk factors include the sheath-to-iliofemoral artery ratio,^19^ moderate-to-severe iliofemoral calcifications, and tortuosity of the iliofemoral artery. Major VCs during TF-TAVR are associated with poor short-term and 1-year outcomes.^20^ A recent study demonstrated that aortic atheroma or a shaggy aorta is associated with periprocedural stroke.^14^ In this study, the criteria defining tough-TF appropriately captured both increased procedural complexity and a higher incidence of VCs. Furthermore, our findings revealed that procedural complexity increased even more when VCs occurred, especially in the tough-TF group. The incidence of major VCs in the tough-TF group was 10.3%, which may be attributed to the single-center design and limited study period. These findings suggest that the tough-TF group may benefit from alternative access strategies. Although the suitability for TC-TAVR was lower than that of the viable-TF group, 86% of patients in the tough-TF group remained suitable for TC-TAVR, indicating a substantial potential to avoid high-risk TF-TAVR.

### Considerations in patients with PAD or on hemodialysis

Careful evaluation of the TAVR approach is warranted in patients with PAD. In the present study, PAD was identified as a negative predictor. The Hostile Registry has shown that facilitated TF-TAVR is effective for relatively simple iliofemoral lesions, whereas alternative access may be more beneficial for complex iliofemoral artery.^21^ Recently, the effectiveness of TF-TAVR facilitated by intravascular lithotripsy (IVL) has been demonstrated in some cases^22^; however, concerns regarding an increased risk of complications remain.^23^

The use of IVL in the carotid artery has been reported both for revascularization^24^ and as an adjunct to TC-TAVR.^25^ In 1 reported case, IVL was used to facilitate TC-TAVR after occlusion of the access-side internal carotid artery, with the IVL catheter was inserted via the femoral artery. In theory, combining carotid interventions with TC-TAVR is feasible. However, this approach requires careful preprocedural planning, including anticipation of potential complications, such as dissection and rupture, and ensuring secure access for device delivery and bailout procedures.

Patients undergoing hemodialysis may be suitable candidates for TC-TAVR, especially when the presence of arteriovenous fistulae preclude trans-subclavian TAVR on the fistulae side. A simulation study of TAVR access in patients on hemodialysis with prior surgical aortic valve replacement demonstrated that the mean CCA diameters were 7.4 ± 0.8 mm on the right and 7.3 ± 0.8 mm on the left. As a result, TC-TAVR was deemed feasible in up to 95.8% of cases. However, limitations included the use of ceCT in only 54% of cases and images with thick slices (8 mm) in 62%, potentially overestimating procedural feasibility.^26^ In our analysis, patients on dialysis were found to be equally suitable for TC-TAVR compared with those not on dialysis, suggesting that they may be appropriate candidates for TC access as a first-line alternative approach.

### Anatomical characteristics of the CCAs

Anatomical differences between the right and left sides may provide insights into the underlying mechanisms influencing TC-TAVR suitability. The diameter of the CCA has been shown to be influenced by male sex, larger body size, advanced age, and hypertension.^27^ However, variations in study populations and the methods used to evaluate carotid artery anatomy have resulted in inconsistent findings. A large-scale study examining the external CCA diameter using ultrasound found no significant differences between the right and left sides.^28^ However, an angiographic study, primarily involving patients with cerebrovascular disease, reported a significantly larger right CCA.^29^ A CT study comparing CCAs in patients undergoing TC-TAVR reported similar minimal diameters of 6.74 ± 0.97 mm on the right and 6.57 ± 1.00 mm on the left. Furthermore, tortuosity was also similar between the sides.^30^

In our cohort, the tough-TF group exhibited a significantly smaller body size and a higher prevalence of atherosclerotic diseases. In this group, factors such as aging and hypertension, rather than body size, likely contributed to increased CCA diameter and increased incidence of atherosclerosis. Although innate vessel diameters were considered comparable between the 2 sides, the right CCA was larger and exhibited greater tortuosity across the cohort. Although the cause for this asymmetry remains unproven, it is speculated that differences in arterial blood flow patterns, the presence of the brachiocephalic artery, and variations in shear stress^31^ may contribute to the right-left differences.

### Implications of tortuosity in TC-TAVR

From a procedural point of view, severe tortuosity may increase the risk of vascular injury during TC-TAVR. Tortuosity of the internal carotid artery has been associated with an increased risk of vascular injury during interventions for acute stroke^32^ and with spontaneous dissection resulting in stroke.^33^^,^^34^ CCA tortuosity was not included among the unsuitability criteria in our study because access may still be feasible depending on vessel characteristics and diameter. Nonetheless, CCA tortuosity should be recognized as a potential risk factor for vascular injury, given its association with stroke or mortality. From this perspective, a vessel with an acceptable diameter and lower tortuosity may provide a rationale selecting left TC-TAVR.

### Study limitations

First, cerebral magnetic resonance imaging was unavailable because of the retrospective nature of the study. Although significant cerebrovascular disease or an inadequate Willis arterial ring may aggravate the suitability for TC-TAVR, recent studies and expert consensus statements do not mandate the assessment of intracranial arteries.^8^^,^^35^ Second, this study did not include patients with aortic stenosis who underwent surgical aortic valve replacement or conservative management; hence, we were unable to identify patients who may eventually require TC-TAVR. Third, factors such as transcatheter heart valve selection, tolerance to general anesthesia, and other feasibility considerations were beyond the scope of this study. Finally, the study was conducted at a single center in Japan, limiting the generalizability of findings to other populations and ethnicities. Future multicenter studies are warranted to address these limitations and validate our findings.

## Conclusions

This study characterized the anatomical features of the CCA in a Japanese population. Overall, the right CCA was larger in diameter but was also associated with increased tortuosity and atherosclerotic plaques, particularly in the tough-TF group. Although TC-TAVR suitability was compromised in the tough-TF group, 86% of patients in this group remained suitable, even within a Japanese cohort with smaller body size. Therefore, TC-TAVR may be a suitable choice for patients requiring an alternative TAVR approach and could contribute to improved clinical outcomes. Further studies are needed to determine the appropriate indications for TC-TAVR and confirm the safety and efficacy of the procedure, particularly in Japanese and other Asian cohorts.

## Funding Support and Author Disclosures

Dr Tada is a clinical proctor for Edwards Lifesciences, Medtronic, and Abbott. All other authors have reported that they have no relationships relevant to the contents of this paper to disclose.
