# Supplementary material for: Suitability for Transcarotid Transcatheter Aortic Valve Replacement in the Japanese Population
Source: JACC Asia. 2026 Mar 3;6(3):345–57. doi: 10.1016/j.jacasi.2025.10.018 (PMC12959277; doi:10.1016/j.jacasi.2025.10.018)
Supplement: Supplemental Tables 1-3 and Supplemental Figures 1-5 [file mmc1.docx]

**Supplemental Materials**Page 2. **Supplemental Table 1**. Association between anatomical findings and access strategy among the tough-TF group

Page 3. **Supplemental Table 2**. Minimum required CCA diameter

Page 4. **Supplemental table 3.** Sensitivity analyses for association between peripheral artery disease and TC-TAVR suitability

Page 5. **Supplemental Figure 1.** Frequency of need for a DrySeal sheath, balloon dilatation, or alternative access according to anatomical factors

Page 6. **Supplemental Figure 2.** Impact of vascular complications on procedural and fluoroscopy time by group

Page 7. **Supplemental Figure 3.** Impact of vascular complications on contrast volume by group

Page 8. **Supplemental Figure 4.** Suitability for TC-TAVR by side

Page 9. **Supplemental Figure 5.** Reasons for unsuitability for TC-TAVR by side

**Supplemental table 1.** Association between anatomical findings and access strategy among the tough-TF group

|  | Overall | TF | | | | | Alternative | |
| --- | --- | --- | --- | --- | --- | --- | --- | --- |
|  |  | TF puncture | | | TF cutdown | | TSC | DAo |
|  |  | Total | DrySeal | Balloon | Total | Balloon |  |  |
| **Anatomical findings** | 117 | 107 | 9 | 12 | 2 | 1 | 7 | 1 |
| Small iliofemoral artery | 54 | 50 |  | 10 | 2 | 1 | 2 |  |
| Heavily calcified iliac artery | 24 | 20 | 1 | 9 | 2 | 1 | 2 |  |
| Tortuous aorta | 25 | 23 | 4 | 1 | 1 |  | 1 |  |
| Shaggy aorta | 38 | 31 | 5 | 2 | 1 |  | 5 | 1 |
| Prior aortic intervention or AA | 9 | 7 |  | 1 |  |  | 2 |  |
| Prior iliac stenting | 3 | 1 |  | 1 |  |  | 2 |  |

The number of cases in which a DrySeal sheath or balloon dilatation was used is shown separately for the TF puncture and TF cutdown groups. Cases in which both techniques were used are counted in both categories. Dryseal : use of a 65-cm DrySeal sheath. Balloon : iliac artery balloon dilatation for device passage. TF, Transfemoral; AA, aortic aneurysm; TSC, trans-subclavian; DAo, direct-aortic.

**Supplemental table 2.** Minimum required CCA diameter

|  |  | Overall | Tough TF | Viable TF |
| --- | --- | --- | --- | --- |
| Minimum required CCA diameter | Applied sizing criteria | N =336 | N =117 | N =219 |
| 5.5 mm | BEV-labeled size  20-mm, 23-mm, 26-mm | 212 (63%) | 75 (22%) | 137 (41%) |
|  | SEV-annulus area  <= 540 mm^2 | 102 (30%) | 40 (12%) | 62 (18%) |
| 6.0 mm | BEV-labeled size  29-mm | 20 (6.0%) | 2 (0.6%) | 18 (5.4%) |
|  | SEV-annulus area  > 540 mm^2 | 2 (0.6%) | 0 | 2 (0.6%) |

The numbers and percentages in the table represent the distribution relative to the entire study cohort (N = 336).

CCA, Common carotid artery; TF, Transfemoral, BEV; Balloon-expandable valve, SEV; Self-expandable valve.

**Supplemental table 3.** Sensitivity analyses for association between peripheral artery disease and TC-TAVR suitability

| Model | Odds ratio | 95%  confidence interval | *p* value |
| --- | --- | --- | --- |
| Primary model | 0.42 | 0.19–0.99 | 0.039 |
| + CKD | 0.41 | 0.18–0.97 | 0.036 |
| + hemodialysis | 0.36 | 0.16–0.88 | 0.020 |
| + IHD | 0.45 | 0.20–1.10 | 0.068 |
| + CVA | 0.43 | 0.19–1.02 | 0.047 |

The primary model was adjusted for age, sex, body surface area, and hypertension. The primary model corresponds to the multivariable model presented in Table 4. Sensitivity analyses were conducted using the primary model with additional individual adjustment for each listed covariate. Odds ratios for peripheral artery disease in each model are shown.

TC, transcarotid; TAVR, transcatheter aortic valve replacement; CKD, chronic kidney disease; IHD, ischemic heart disease; CVA, cerebrovascular accident.

**
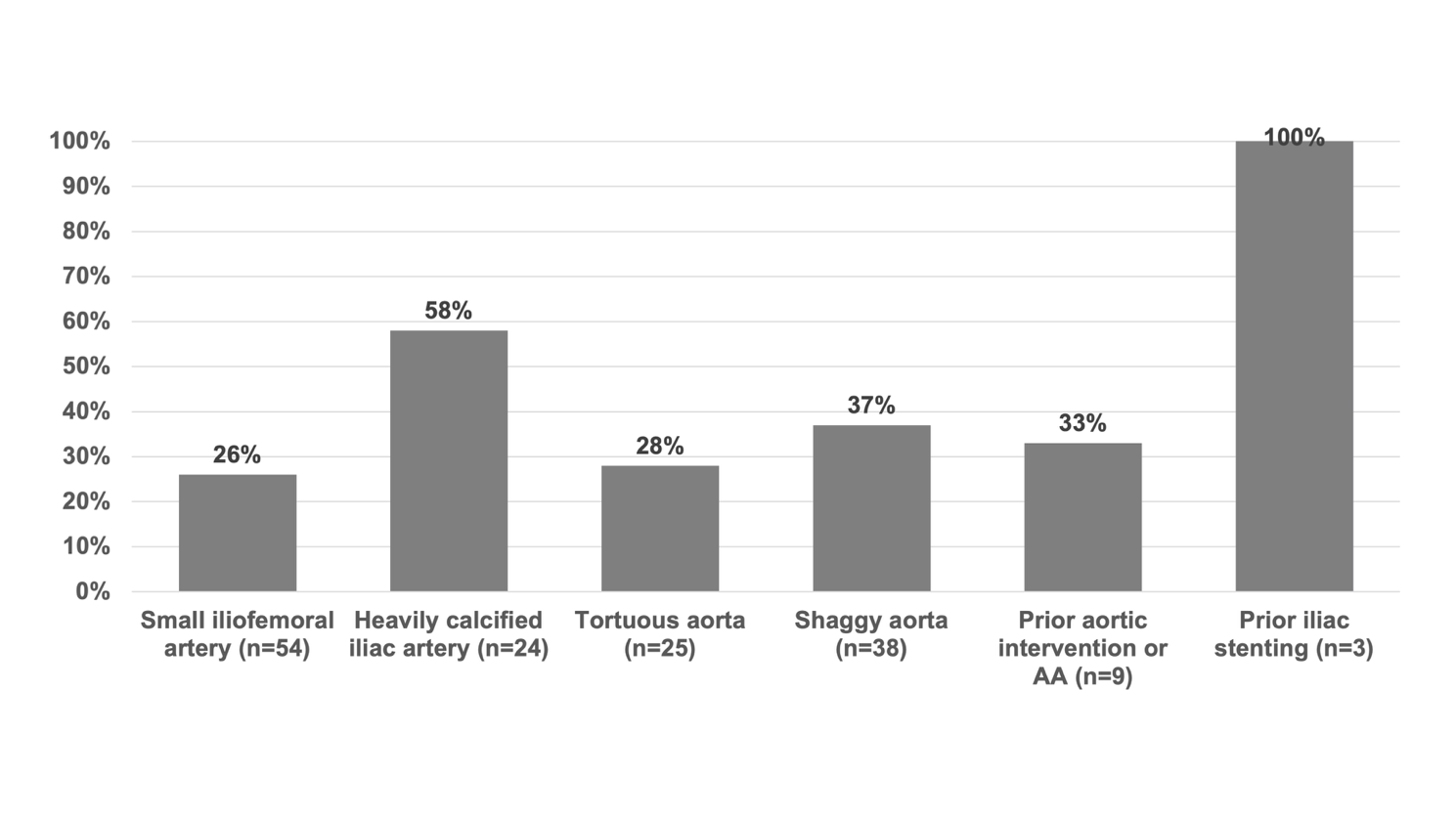
Supplemental Figure 1.** Frequency of need for a DrySeal sheath, balloon dilatation, or alternative access according to anatomical factors

Percentages within each bar indicate the proportion of cases requiring a 65-cm DrySeal sheath, iliac artery balloon dilatation for device passage, or an alternative access route.

AA, aortic aneurysm.

**
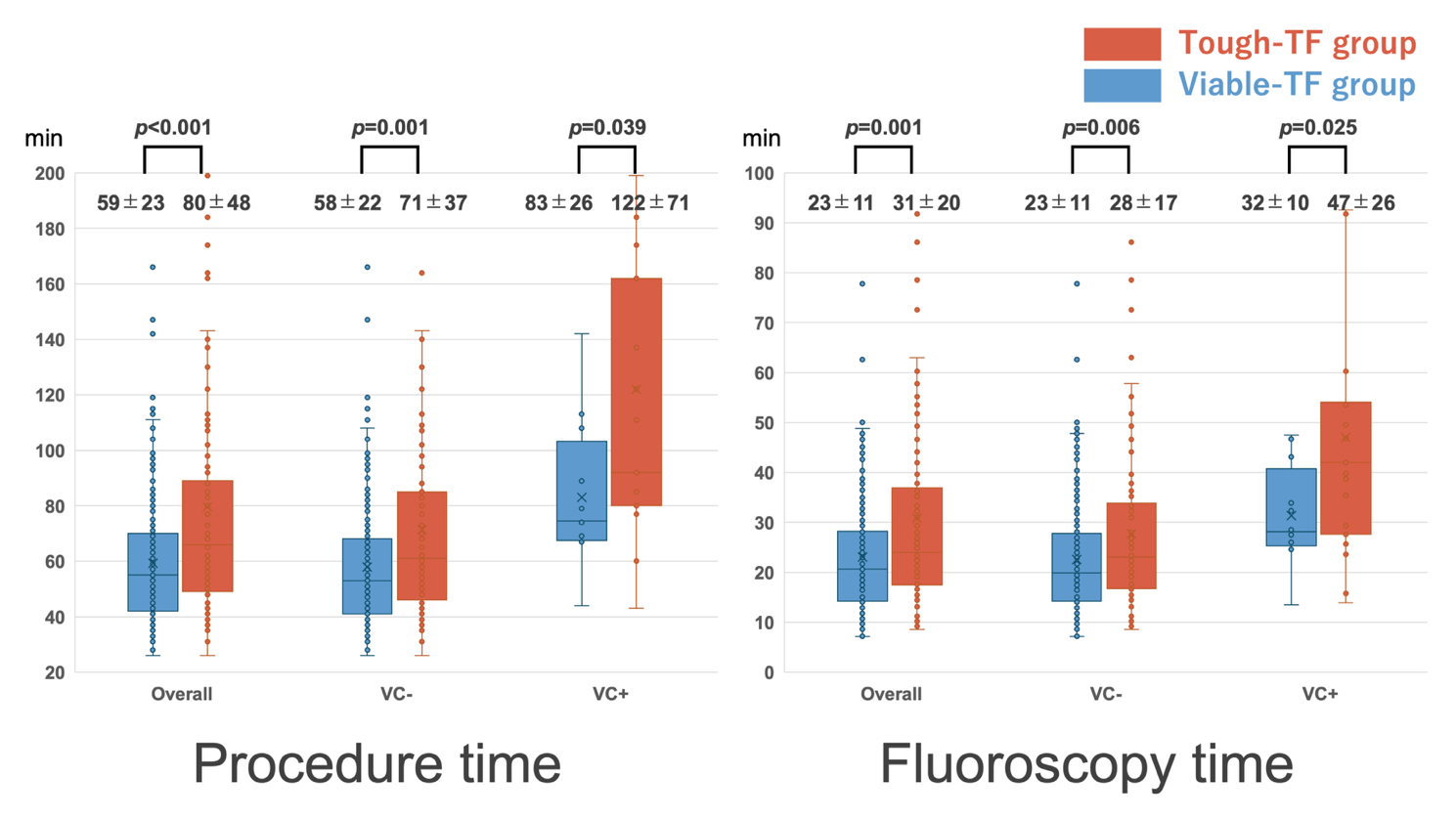
Supplemental Figure 2.** Impact of vascular complications on procedural and fluoroscopy time by group

Both procedural and fluoroscopy times were significantly longer in the tough-TF group, irrespective of vascular complications. When vascular complications occurred, these times increased markedly in the tough-TF group, suggesting that this group is inherently associated with greater procedural complexity and that bailout procedures impose an additional burden on these patients.

TF, transfemoral; VC, vascular complications.

**
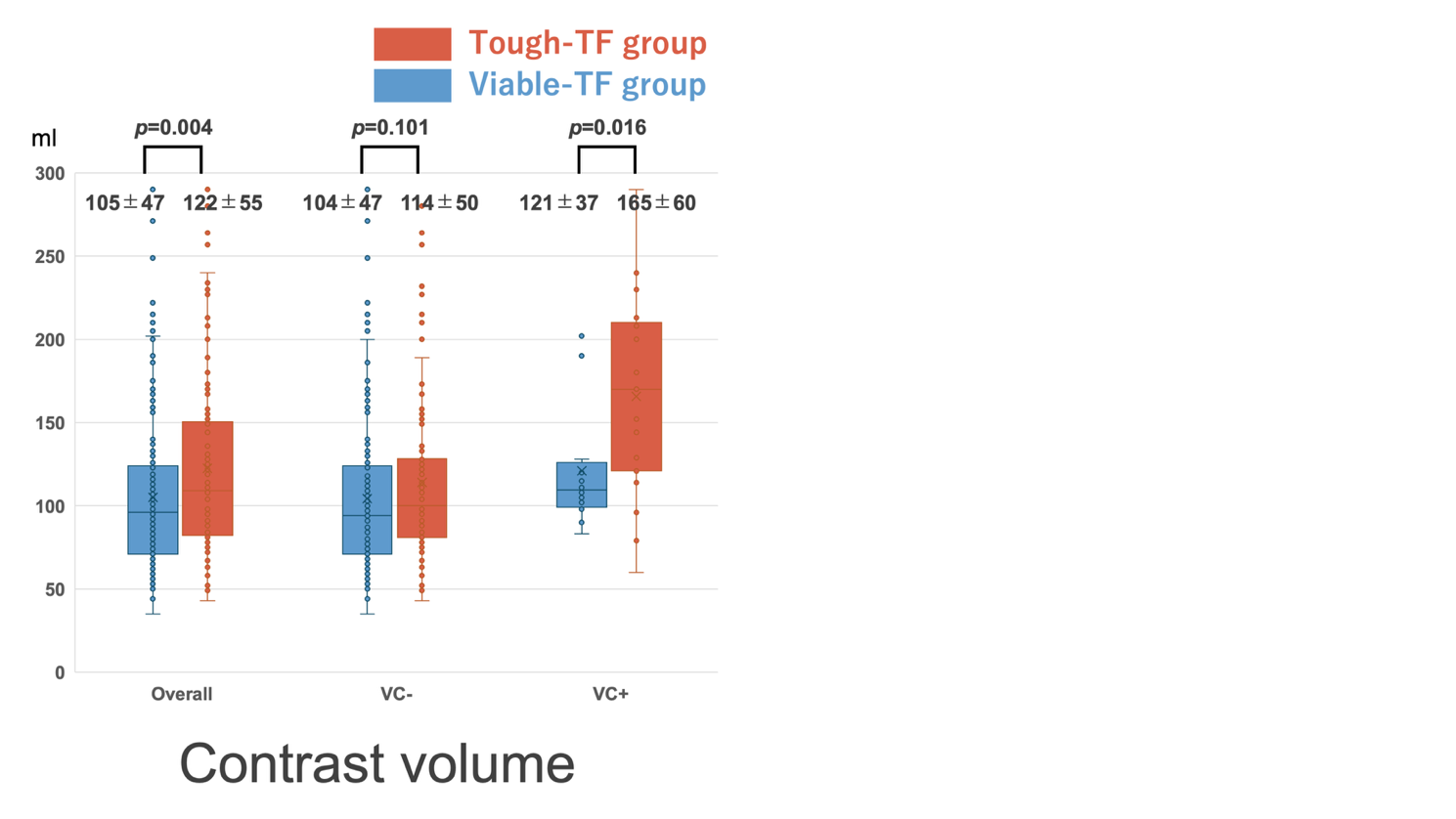
Supplemental Figure 3.** Impact of vascular complications on contrast volume by group

Overall contrast volume was higher in the tough-TF group, driven by the increased volume observed in cases with vascular complications.

TF, transfemoral; VC, vascular complications.

**
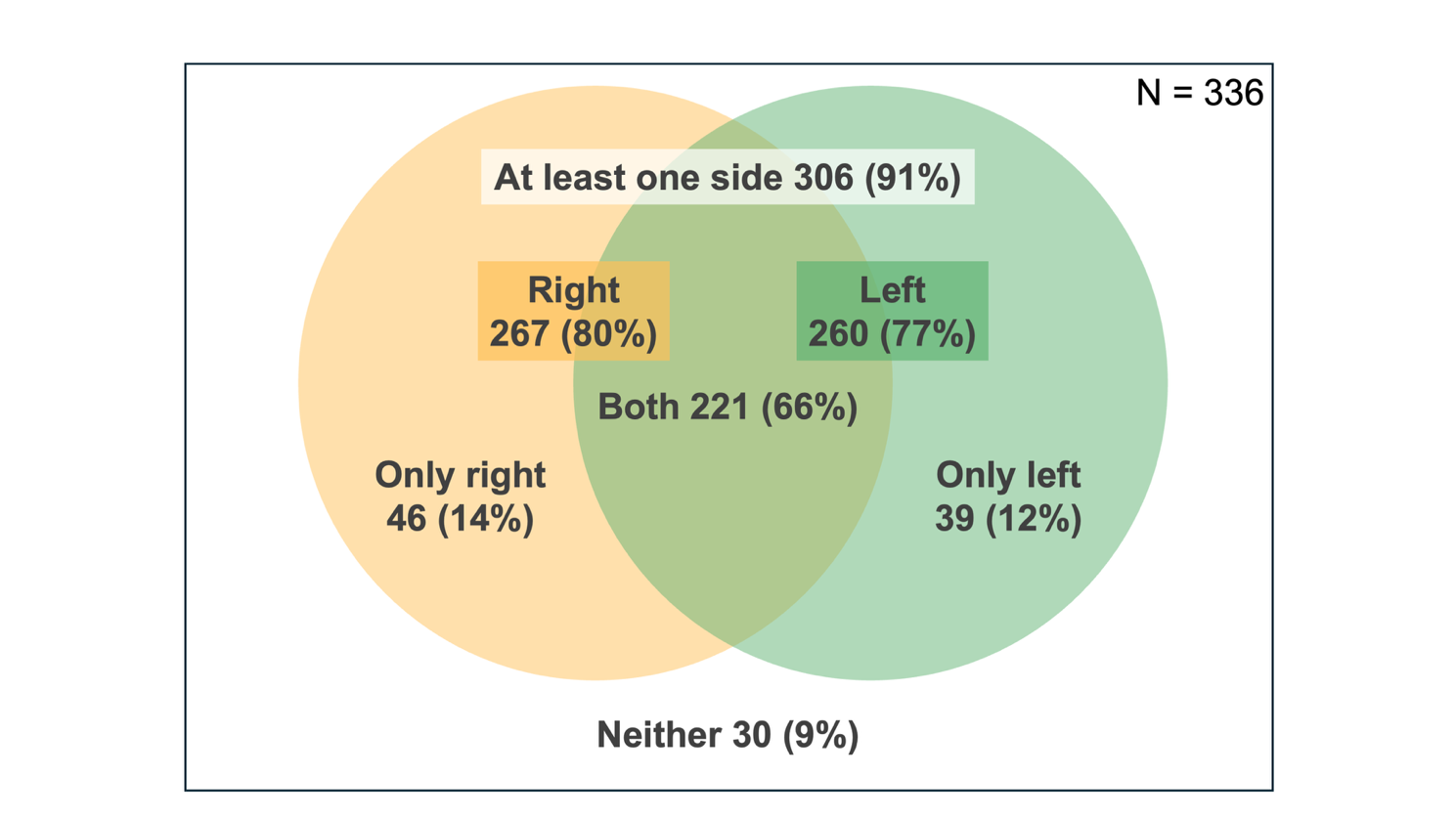
**

**Supplemental Figure 4.** Suitability for TC-TAVR by side

The Venn diagram depicts suitability for TC-TAVR across the study cohort. 306 patients (91%) were suitable on at least one side.

TAVR, transcatheter aortic valve replacement; TC, transcarotid.

**
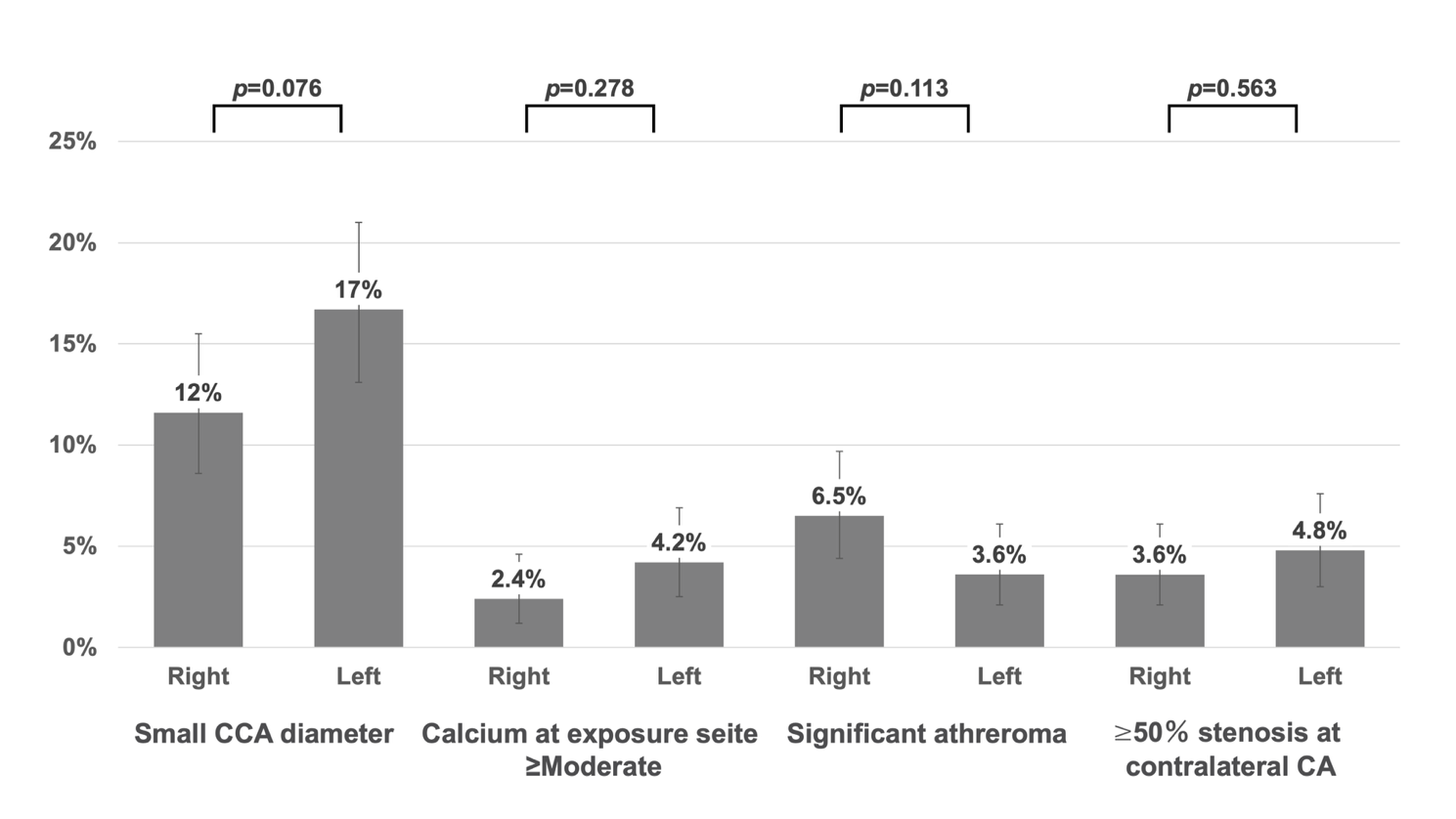
**

**Supplemental Figure 5.** Reasons for unsuitability for TC-TAVR by side

In the overall cohort, small vessel diameter was the primary reason for unsuitability (12% vs. 17%, p = 0.076), while other factors contributed less frequently.

TAVR, transcatheter aortic valve replacement; TC, transcarotid.
